# Reprogrammable meta-hologram for optical encryption

**DOI:** 10.1038/s41467-020-19312-9

**Published:** 2020-10-30

**Authors:** Geyang Qu, Wenhong Yang, Qinghai Song, Yilin Liu, Cheng-Wei Qiu, Jiecai Han, Din-Ping Tsai, Shumin Xiao

**Affiliations:** 1 Ministry of Industry and Information Technology Key Lab of Micro-Nano Optoelectronic Information System, Shenzhen Graduate School, Harbin Institute of Technology, 518055 Shenzhen, China; 2 Collaborative Innovation Center of Extreme Optics, Shanxi University, 030006 Taiyuan, China; 3 Department of Electrical and Computer Engineering, National University of Singapore, 4 Engineering Drive 3, Singapore, 117583 Singapore; 4 National Key Laboratory of Science and Technology on Advanced Composites in Special Environments, Harbin Institute of Technology, 150080 Harbin, China; 5 Department of Electronic and Information Engineering, The Hong Kong Polytechnic University, Hong Kong, China

**Keywords:** Applied optics, Metamaterials

## Abstract

Meta-holographic encryption is a potentially important technique for information security. Despite rapid progresses in multi-tasked meta-holograms, the number of information channels available in metasurfaces is limited, making meta-holographic encryption vulnerable to some attacking algorithms. Herein, we demonstrate a re-programmable metasurface that can produce arbitrary holographic images for optical encryption. The encrypted information is divided into two matrices. These two matrices are imposed to the incident light and the metasurface, respectively. While the all-dielectric metasurface is static, the phase matrix of incident light provides additional degrees of freedom to precisely control the eventual functions at will. With a single Si metasurface, arbitrary holographic images and videos have been transported and decrypted. We hope that this work paves a more promising way to optical information encryption and authentication.

## Abstract (web summary)

Here, the authors demonstrate a re-programmable metasurface that can produce arbitrary holographic images for optical encryption. The encrypted information is divided into two matrices and defined to the incident laser to produce arbitrary holographic images.

## Introduction

In modern society, almost everything is closely related to the internet currently or in the near future. The recent reports of attacks by hackers on the databases show that the information security and authentication are more challenging, especially for the large commercial and financial companies^1^. In past decade, many types of optical encryption techniques based on classic and quantum optics have been developed to achieve reliable security of information^2,3^. Owing to its compact and non-replicable nature, metasurface-based hologram has gradually been a promising approach^4–10^. Metasurface is a kind of two-dimensional materials that has the capability of precisely and fully controlling the incident light^11^. By carefully designing the response of each antenna to different polarization, incident angle, wavelength, or orbital angular momentum (OAM), multiple holographic images have been successfully implemented into a single metasurface in the past few years, significantly expanding the capacity and security of information encryption^7,12–19^. Despite of these progresses, the demonstrated meta-holograms are either static or binary^4–20^. While different multiplexing techniques based on spatial, polarization, wavelength, and OAM have been combined recently, the number of information channels is still finite and the current record value is only ~2^6^ (refs. ^7,15–26^). Similar to the double-random-phase encryption technique, the finite channel number makes the meta-holographic encryption vulnerable to certain attacks because the key for decryption has to be used repeatedly without frequent update^27^. Therefore, from the point view of information security, it is highly desirable and urgent to realize a meta-hologram with infinite channels and higher security. Here, we revisit the light–metasurface interaction and experimentally demonstrate a reprogrammable meta-hologram for optical security and authentication.

## Results

### Working principle

**Fig. 1:**
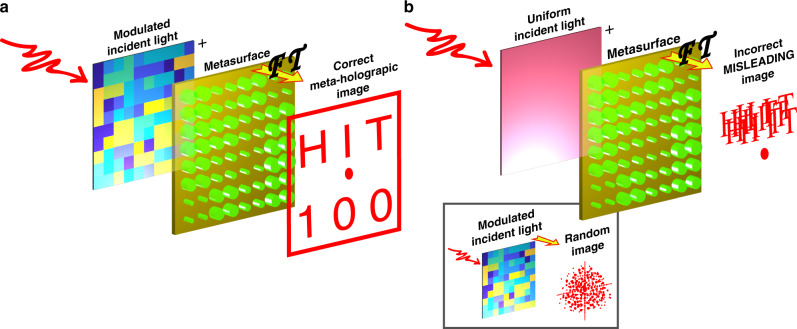
The working principle of reprogrammable meta-hologram. **a** The schematic of the meta-holographic image with the modulated incident beam. **b** The holographic image of metasurface when it is illuminated with an incorrect uniform laser beam. The inset shows that the incident beam itself cannot provide correct information.

In principle, the interaction between light and metasurface can be expressed as^28^:

1 $$U(x,y,z) = {\int\!\!\!\!\!\int} {U_{{\mathrm{inc}}}(x_0,y_0)U_{{\mathrm{meta}}}(x_0,y_0)h(x - x_0,y - y_0,z)} dx_0dy_0,$$

where *U*_inc_(*x*_0_, *y*_0_) and *U*_meta_(*x*_0_, *y*_0_) correspond to the functions of incident light and metasurface, *h*(*x*, *y*, *z*) is an impulse response. The conventional approaches are mostly focusing on the response of nanoantennas in metasurface^4–19^. Only few parameters of the incident light have been considered, e.g., polarization and OAM etc.^7,15–26^. Since the metasurface is typically static, the information channel is thus determined by the degrees of incident light and is strongly limited^7,15–26^. In this research, we divide the required phase profile of a holographic image into two matrices. One matrix is imposed to the metasurface (*U*_meta_(*x*_0_, *y*_0_)) as before, the other one is defined on the incident beam (*U*_inc_(*x*_0_, *y*_0_)). This assumption is not only valid for algorithm but also consistent with the modern optical security system, which consists of light source, lens, detectors, phase only masks, and spatial light modulators (SLMs) etc.^29,30^. On one hand, the two-dimensional matrix of incident beam can significantly expand the number of information channels. Even though the metasurface is static, the large matrix of incident beam still has enough large parameters to alter the output beam (*U*(*x*, *y*, *z*)) and the corresponding holographic images. On the other hand, the split information in transported light and metasurface can improve the security. The codes for the modulation of incident light is transported through the internet, whereas the metasurface can be implemented into the terminal decrypted devices. In case that the transported codes are cracked, they can only produce a random beam without any useful information (see inset in Fig. 1b). If the metasurface is stolen and illuminated with an incorrect beam, no information can be hacked or a misleading image could be shown up (Fig. 1b). Only when the modulated incident light matches the metasurface, as shown in Fig. 1a, the encrypted image can be displayed. According to Eq. (1), for encrypted information with fixed overall matrix, the matrices for the incident light and the metasurface can be separated arbitrarily. Consequently, the roles of two matrices can be interchanged and the flexibility of the system has been dramatically increased (see Supplementary Fig. 2).

### Experiments

**Fig. 2:**
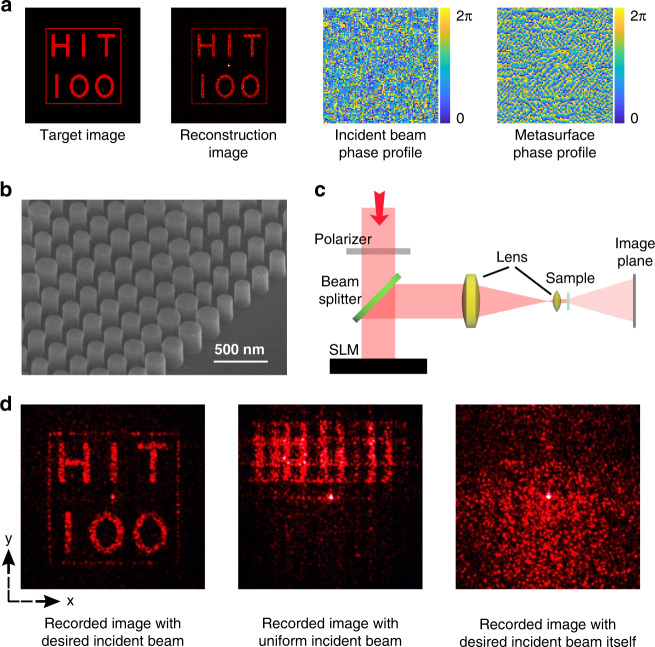
The experimental demonstration of meta-hologram. **a** The designed image, the simulated holographic image, and two separated phase profiles. **b** The top-view SEM of the Si metasurface. **c** Schematic for capturing the meta-hologram. **d** The recorded meta-holographic images with the designed incident beam (left), with a uniform laser beam (middle), and the image of incident laser itself (right). Here the lattice size is *L* = 240 nm.

To test the above analysis, we have numerically designed the reprogrammable meta-hologram^31,32^. Taking an image of “HIT 100 (100th anniversary of Harbin Institute of Technology)”as an example, the required phase profile for a holographic image has been calculated based on Gerchberg–Saxton algorithm. After considering both of the efficiency and the fabrication difficulty, the continuous phase profile is discretized into eight-level phase steps (Supplementary Fig. 3). Then the discretized matrix is separated into two matrices, which are imposed on the incident beam and the nanoantennas of Si metasurface, respectively (Fig. 2a). The phase profile of incident light is controlled by a SLM (UPOLabs-HDSLM80R). The phase only metasurface is fabricated with standard silicon on sapphire wafer via a combined process of electron-beam lithography and reactive ion etching (Supplementary Notes 1–3)^32^. Figure 2b shows the top-view scanning electron microscope (SEM) image of the metasurface. It is composed as Si nanopillars with different radius *R*. The height of Si nanopillar is fixed at 230 nm. In Fig. 2b, eight types of nanopillars with *R* = 44, 58, 64, 68, 73, 78, 85, and 95 nm are employed to provide relative high transmittance, and abrupt phases from 0 to 7π/4, simultaneously (Supplementary Notes 1–3). In this experiment, each phase pixel of Si metasurface in Fig. 2b consists of multiple nanopillars with the same size. This setting is designed to fit the pixel size of Si metasurface and the matrix size of incident light.

The Si metasurface is optically characterized with the setup in Fig. 2c. Basically, the linearly polarized laser is generated with a He–Ne laser (633 nm) and a linear polarizer. The light is modulated by the SLM before it is focused onto the sample via a 4f system. The transmitted hologram is projected onto a screen in the far field and captured with a CCD camera (see the “Methods” section). Figure 2d shows the image of intensity distribution generated by the metasurface. Similar to the numerical calculation, a “HIT 100” image has been experimentally produced when the Si metasurface is illuminated by the laser beam with the designed phase profile. The calibrated conversion efficiency of the meta-hologram is ~67% (Supplementary Fig. 8). For a direct comparison, the same Si metasurface has also been illuminated with a uniform laser beam. As depicted in the middle panel of Fig. 2d, a misleading pattern with incorrect information is achieved. Similar encryption also holds true for the light source only. The modulated laser beam itself is also an uniform laser spot with speckles instead of the designed information (right panel in Fig. 2d).

**Fig. 3:**
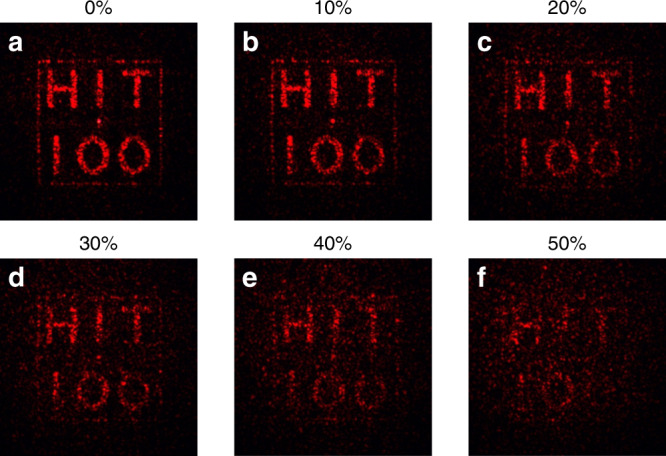
The vulnerable test of the meta-hologram. From **a**–**f**, the mismatch ratio of the incident light increases from 0 to 50% with a step of 10%.

The above experiments show that the encrypted information can be protected even though the key or the metasurface was stolen. For a better demonstration, the vulnerability of the optical encryption scheme has also been tested with a different key mismatch ratio. Figure 3 summarizes the experimental results as a function of the modification on incident beam. It is clear to see that the meta-holographic image becomes blurry when the mismatch ratio is >10%. In case of the mismatch ratio exceeds 40%, the “HIT 100” image is close to the noise level and hard to be identified. This means that the eavesdropper has to steal ~60% of the incident matrix and the terminal devices, simultaneously to barely recover the encrypted information. This is even higher than the recently demonstrated metasurface-based ghost imaging algorithm^8^. Note that the above case is valid for the situation that both of the initial positions ((0,0) point of two matrices and the longitudinal deviation from the focal point) and pixel sizes are known, and matched one another. Without the above information, it shall consume orders of magnitude longer time to randomly test the right key for decryption (Supplementary Figs. 6 and 7).

**Fig. 4:**
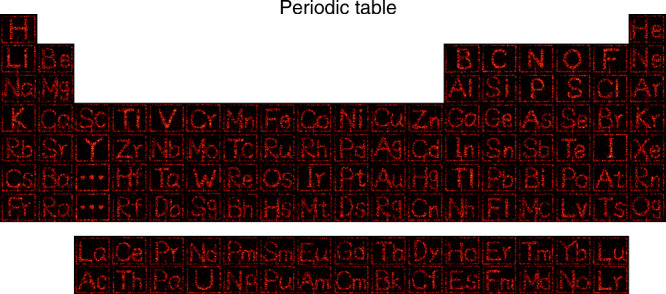
The reprogrammable meta-hologram. The holographic images of periodic element table. Each element in this table is one holographic image that is generated with the same metasurface in Fig. 2.

In principle, the right key for decryption can still be hacked if the random test time is infinitely long^27^. To avoid the potential attack, the key must be updated frequently. As a result, the reprogrammable metasurface is highly desirable for the applications of optical encryption. According to Eq. (1), even though *U*_meta_(*x*_0_, *y*_0_) is fixed and known, the displayed holographic image *U*(*x*, *y*, *z*) can still be changed by controlling the incident light *U*_inc_(*x*_0_, *y*_0_). Then the meta-hologram becomes all-optically controllable. In this sense, although the terminal hardware, such as Si metasurfaces are hard to be tuned in pixel level, the precise control of the incident beam can still frequently update the keys for decryption. Figure 4 summarizes the experimental demonstration of the all-optically reprogrammable meta-hologram. In this experiment, the Si metasurface is kept as the same one as Fig. 2b. By only modifying the phase distributions of the incident light, the displayed holographic image becomes an image of hydrogen element “H”, which is totally different from the image of “HIT 100” in Fig. 2. With the further modification on incident light, the displayed hologram can be switched to an image of hydrogen element or any other elements. With the continuous change of incident light, we have experimentally realized the holographic images of the entire periodic table of the elements, with 118 types of elements. Here, each element is an individual holographic image produced by the same metasurface. The periodic table of elements is a clear demonstration of the programmable and all-optically controllable meta-hologram.

We note that the number of periodic element table is not the limit of the information channel of our design. With the same Si metasurface, we basically can realize arbitrarily shaped holographic images, including the holographic image with complicated contents (Supplementary Fig. 10). To verify this possibility, we have also produced a set of holographic images by modulating the incident beam and plotted them in Supplementary Fig. 11. Each panel corresponds to one frame of a pinball classic game. By playing these frames sequentially, a video of the pinball game from start to the end has also been achieved (Supplementary Movie 1). These results further demonstrate the potential of the proposed technique in the transportation of videos as well, significantly expanding the potential applications of metasurface holograms.

## Discussion

In summary, we have revisited the light–metasurface interaction and experimentally realized the reprogrammable meta-hologram. By separating the designed phase profile to two matrices, and defining them to incident laser and metasurface, we find that the lack of pixel-level control of metasurface in optical frequency range can be made up via the all-optical control. With the phase control of incident beam, we have experimentally demonstrated that a single metasurface can display arbitrary holographic images, as well as encrypted videos. This technique shall find its applications in information security and authentication. It also paves a promising step toward dynamic displays as well.

## Methods

### The numerical simulation

The silicon metasurface was numerically simulated with a commercial software COMSOL Multiphysics. The Si nanorods are placed on a sapphire substrate with refractive indices of 3.88 and 1.76 at 633 nm. The height of nanorod was kept at 230 nm. The radius of nanorod and the lattice size are *R* and *L*, respectively. Periodic boundary conditions were applied in horizontal direction to mimic the periodic structure, and perfectly matched layers were used to absorb the transmission and reflection waves. The details are shown in Supplementary Notes 1 and 2.

### The fabrication of Si metasurface

The Si metasurfaces were fabricated by a combined process of electron-beam lithography and reactive ion etching. The 230 nm single-crystal silicon film on a sapphire wafer was cleaned with acetone and coated with 100 nm electron-beam resist polymethyl methacrylate. The sample was baked at 180 °C for 1 h and then patterned by electron-beam writer (Raith E-line) with a dose 90 µC/cm^2^ under an acceleration voltage 30 kV. After developing with MIBK&IPA (1:3) solution, 15 nm Cr was deposited on the sample using E-beam evaporation (SKE_A_75), and Cr hard mask was realized via a liftoff process in remover PG solution (Micro Chem) for 24 h. The pattern was transferred to the Si through reactive ion etching with SF_6_ and CHF_3_ in Oxford Plasmalab System 100. The vacuum degree was 10^−5^ and the gas flow was 5 and 50 sccm for SF6 and CHF3, respectively. Finally, the Si metasurfaces were obtained by removing the Cr mask in Cr etchant solution (Aldrich Chemistry) for 30 min.

### The optical characterization

During the optical characterization, the alignment of the phases in incident beam the phases in metasurface is crucial. The light is modulated by the SLM before it is focused onto the sample via a 4f system. The transmitted hologram is projected onto a screen in the far field and captured with a CCD camera. The SLM is switched to intensity module to clearly seen the focused beam, and precisely match it with the metasurface. Then it is switched back to phase module and measure the meta-hologram.

## Supplementary information

Supplementary Information

Description of Additional Supplementary Files

Supplementary Movie 1

## Data Availability

The data that support the findings of this study are available on request from the corresponding authors.
