# Supplementary material for: Reprogrammable meta-hologram for optical encryption
Source: Nat Commun. 2020 Oct 30;11:5484. doi: 10.1038/s41467-020-19312-9 (PMC7603497; doi:10.1038/s41467-020-19312-9)
Supplement: Supplementary file 1 — Supplementary Information [file 41467_2020_19312_MOESM1_ESM.pdf]

Supplemental information for:

Reprogrammable meta-hologram for optical encryption

Geyang Qu[1], Wenhong Yang[1], Qinghai Song[1,2], Yilin Liu[1], Cheng-Wei Qiu[3], Jiecai Han[4], Din Ping Tsai[5,*], Shumin Xiao[1,2,4,*]

1. Ministry of Industry and Information Technology Key Lab of Micro-Nano Optoelectronic Information System, Shenzhen Graduate School, Harbin Institute of Technology, Shenzhen, 518055, China.

2. Collaborative Innovation Center of Extreme Optics, Shanxi University, Taiyuan 030006 China.

3. Department of Electrical and Computer Engineering, National University of Singapore, 4 Engineering Drive 3, Singapore 117583, Singapore.

4. National Key Laboratory of Science and Technology on Advanced Composites in Special Environments, Harbin Institute of Technology, Harbin 150080, P. R. China.

5. Department of Electronic and Information Engineering, The Hong Kong Polytechnic University, Hong Kong

Emails: *dinping.tsai@polyu.edu.hk, *shumin.xiao@hit.edu.cn

Based on the fact that spatial light modulator (SLM) is frequently used in optical encryption, we have designed and demonstrated experimentally that the combination of the all-dielectric metasurface and the modulation on incident beam can significantly expand the potentials of both optical encryption and optical metasurfaces in the main manuscript. In this supplemental information, we show all the

experimental details about the sample design, fabrication, and optical characterization. Additional information to support the optical security and the ability of transporting video have also been demonstrated.

Supplementary Note 1: The design and characterization of reprogrammable metasurface

The phase profile for Si metasurface and the SLM

The reprogrammable metasurface is designed via two steps. The first step is the calculation of required phase profile for the designed image. This is realized by the conventional Gerchberg-Saxton (GS) algorithm followed by the iteration of optimization. The optimization is applied to improve the efficiency and the signal-to-noise contrast. After achieving the phase profile for the designed image, we separate the incident phase profile into two matrices with the same dimensions (see Supplementary Figure 1(a)). One matrix is applied onto the SLM, whereas the other one is defined for the Si metasurface.

The design in Supplementary Figure 1a has intrinsically advantages in all-optically controllable metasurface. Although the phases in Si metasurfaces cannot be tuned, the phase profiles for different images can still be constructed by tuning the phase distribution of the incident laser. As shown in Supplementary Figure 1b, without changing the phase profiles for Si metasurface, different images such as HIT, 100, and META can be re-constructed by modifying the phase distribution of SLM.

The two matrices for the metasurface and the SLM are selected as the following. The phase profile for a meta-hologram was calculated with the G-S algorithm and discretized into a matrix φ_{GS}. In principle, φ_{GS} can be the sum of two arbitrary matrices with the same dimensions. Then we pick a randomly designed phase profile of φ_{meta}, which is defined to the metasurface. The phase for the SLM, φ_{SLM}, can be simply achieved from the equation $\varphi_{SLM} = \varphi_{GS} - \varphi_{meta}$ and endowed to the SLM. Note there is no special requirements for the phase profile of φ_{meta}. It can be randomly selected. This setting has two advantages. For any desirable hologram, it can always be produced with the same metasurface by changing the phase φ_{SLM}. This is the fundamental basis of transmitting the video. Meanwhile, by carefully designing the metasurface, the information for the incident light can carry the mis-guiding information. As depicted in Supplementary Figure 2, the information for SLM carries the intensity distribution as well as phase profile. In case that the information for SLM was stolen, the hacker shall get the wrong QR codes and guided to a different position. Only both of the metasurface and φ_{SLM} are known, the encoded QR codes can be decrypted.

As the sizes of Si nano-antenna is much smaller than the diffraction limit, 4×4 matrix of Si nano-antennas are applied to match the pixel size of incident laser beam. Meanwhile, for simplicity, the phases in Si metasurface are 0, $\pi/4$, $\pi/2$, $3\pi/4$, π, $5\pi/4$, $3\pi/2$, and $7\pi/4$. The 8-level phase profile is selected by considering both of the fabrication difficulty and the efficiency of wavefront

engineering. The results are shown in Supplementary Figure 3. It is easy to see that the efficiency increases dramatically with the number of phase level increasing. For the 8-level phase profile, the calculated efficiency is over 80%. While the efficiency for 16 or higher level phase profile is a little bit larger, the corresponding fabrication is more difficult because the difference between nano-antennas becomes smaller. Therefore, we select the 8-level phase profile for the design and experimental realization of Si metasurface.

The numerical simulation of Si metasurface

After determining the phase distribution for Si metasurface, such as phase profile must be realized with Si nano-antennas. In this research, the Si nano-antennas are simulated with a commercial software (COMSOL Multiphysics). To minimize the influence of polarization, we select the Si nanopillar with circular cross-section as the fundamental element. As depicted in Supplementary Figure 4a, the Si nanopillars are placed on a sapphire substrate. The radius and lattice size are R and L, respectively. The height of Si nanopillar is fixed at 230 nm. The silicon and sapphire substrates are both single crystals with refractive indices of 3.88 and 1.76 at the operating wavelength. Supplementary Figure 4b shows the transmission spectrum of Si nanopillar array with R = 68 nm and L = 240 nm. A clear resonant dip can be seen around 633 nm. The dashed line in Supplementary Figure 4b is the corresponding phase change at the nanopillar. With the changes of radius R and lattice size L, the resonant wavelength shifts correspondingly.

Based on the above analysis, for a fixed wavelength, the Si nanopillar with different size can provide different phase change. Supplementary Figure 4c and Supplementary Figure 4d are the transmission and phase change at 633 nm as a function of radius R and lattice size L. A clear region with both of high transmission and phase change from 0 to 2π can be achieved. Within this region, we have selected 8 nanopillars to generate the designed metasurfaces. Note that circular nano-antenna is insensitive to the polarization. It works well for linear, circular and unpolarized light as well. The requirement of linear polarization in main text comes from the limitation of SLM in experiment. This can be solved by applying polarization insensitive SLM.

The fabrication of Si metasurface

In experiments, the Si metasurfaces are fabricated with a top-down process which is a combination of electron-beam lithography and reactive ion etching. All the samples are fabricated with a 230 nm single crystalline Si film on a sapphire substrate. The whole process contains 7 steps that are schematically shown in Supplementary Figure 5 below. At the beginning, the Si film wafer is cleaned with acetone and ultrasonic. Then 100 nm PMMA electron beam resist is spin-coated onto the top-surface. After spin-coating, the sample is baked at 180°C for 1 h. Then the inversed nanostructures are patterned onto the PMMA resist. The dose is 90 $\mu C/cm^2$ and the acceleration voltage is 30 kV. After electron beam exposure, the exposed PMMA are formed by development in MIBK:IPA solution with a mixture ratio 1:3.

Basically, 100 nm PMMA is good for achieving small feature sizes. But it is too thin for the reactive ion etching process. In our experiment, 15 nm Cr layer is evaporated onto the nanostructure within an electron beam evaporator. The vacuum is reduced to 2×10^{-7} and the evaporation speed is 0.2 Å/s. After cooling to room temperature, the sample is placed into remover-PG solution for 24 hours. The PMMA resist is dissolved and the Cr nanostructures are thus produced. Then the Cr nanostructures are used as hard mask in the reactive ion etching process. The Si film is etched in Oxford 80 RIE with SF_6 and CHF_3. The gas flows are 5 sccm and 50 sccm, respectively. After 10 s etching in Oxford 80 RIE, 230 nm Si layer is etched to the sapphire substrate with a nearly perfect vertical sidewall. Finally, the sample is achieved by placing it in Cr etching solution to remove the hard mask and cleaning it with deionized water.

The phase modulation of incident laser

In additional to the Si metasurface, it is also important to impose the phase information to the incident laser and focus it to the same size as the silicon metasurface. The polarization of incident He-Ne laser is tuned to the designed working direction of SLM. The required phase profile is provided by the rotation of liquid crystal molecules in each pixel of the SLM. The modulated laser is then focused by an optical lens of 15 cm and re-collimated with a 60X objective lens before it illuminates onto the Si metasurface with the designed beam size. The meta-hologram is thus produced in the transmission direction and collected by an

objective lens and another optical lens.

The alignment of the incident phase profile to the metasurface

As we claimed in the main text, a 40% mismatch between phase planes of metasurface and SLM will completely destroy the holographic image. In order to generate a clear hologram image with high efficiency, it is very important to match the incident phase profile and the metasurface phase profile. There are several steps to achieve a perfect alignment. First of all, the SLM is switched to the intensity module. Compared with the phase profile, the distributions and changes in intensity are much easier to be seen with CCD camera. Based on this visuality and the pixel-level control of SLM, the size, position and direction of the focused beam light can be precisely tuned to match the metasurface. Then the SLM can be switched to the phase module and the designed functions can be displayed by setting the corresponding phase profile for the incident light.

Supplementary Note 2: The optical characterizations

The results of meta-hologram with un-matched initial position

In the main text, we have shown the optical setup for the characterization of meta-hologram. During the vulnerability test, we show that the encoded information can only be obtained when more than 60% keys have been hacked. Here we show that the security for our design can be even better. As illustrated in Supplementary Figure 1, the required phase profile can be separated into two matrices. The designed image

can be displayed only when two matrices match one another. The fundamental basis for the experiment is the 0,0 points of two matrices at the same position. In real case, the hacker won't know this information at all.

In the optical experiment, the incident light is controlled by a spatial light modulator. The device used in our experiment has the switching time of 1s, giving a refreshing rate of 60 Hz. The device works well for a temperature range from 20°C to 50°C. Note that all these performances can be further improved. In principle, the switching time can be on the order of micro-second and the working temperature range can also be better.

In Supplementary Figure 6a, we show the experimental results of meta-hologram as a function of position deviation. With the deviation between the focused incident laser and the Si metasurface, we can see that the displayed meta-hologram "HIT 100" quickly degrades with a lateral shift less than 1.4 micrometer. Similar experiments have also been done with the rotation between two matrices. As depicted in Supplementary Figure 6b, the meta-hologram "HIT 100" also rapidly decreases with a small rotation of a few degree. This information is important for the improvement of optical security. Without the information of 0,0 point of both incident matrix and Si metasurface, it is almost impossible to know that the noise level speckle pattern is caused by the imperfect alignment or the wrong locations.

In additional to the lateral sensitivity, the longitudinal deviation can also be applied to improve the security of the system. By fixing the rest conditions, we have

studied the displayed holographic image as a function of the longitudinal deviation. All the results are displayed in Supplementary Figure 7. The holographic image of 'W' deteriorates quickly when the sample is placed out of the focal point. When the metasurface is 7 micrometers away from the optimized position, the hologram degrades to the noise level. Such kind of dependence can also be understood with the Equation in the main text. The size of light beam increases out of focus and mismatch the size of metasurface, quickly reducing the quality of holographic images. Therefore, the combination of the sensitivity to lateral, orientation angle and longitudinal mismatch, out system can have a much better security. Even if the hacker has known the information of both metasurface and incident light profile, he/she still need to know an exact position where two matrices will match to each other in the 3-dimensional space.

The efficiency of the meta-hologram

For the applications of meta-hologram, the conversion efficiency is very critical. The numerical calculation shows that the efficiency for 8-level phase profile can be above 80%. Experimentally, the presence of surface roughness and phase deviation will degrade such efficiency. In our experiment, we have measured the efficiencies of a series of meta-holograms. All the experimental results are shown in Supplementary Figure 8. For different holographic images, the efficiencies are always around 67% with a small variation of a few percent.

The phase profiles of incident laser for the periodic table of element

In the main manuscript, we have experimentally shown that the same metasurface can realize more than 118 different holograms of elements (see Fig. 9). As the theoretical model depicted in Supplementary Figure 1, these meta-holograms are produced by modulating the phase profile within the incident laser. Here we show the calculated phase profiles of incident lasers for the 118 elements in periodic table of elements (see Supplementary Figure 9).

The holographic images of Schrödinger equation

In the main text and SI, we have demonstrated that the reprogrammable metasurface has the ability of producing multiple holographic images with the same metasurface. Here we further show that the function of our reprogrammable metasurface is not limited to the simple cases. It is also applicable to complicated contents. To demonstrate this possibility, we have designed a Schrödinger equation as shown in Supplementary Figure 10a. With the same metasurface as the main text, we have also experimentally proved this capability. The results are shown in Supplementary Figure 10b. It is very close to the numerical results and clearly demonstrating the potential of our design in complicated images. We note similar capability has also been demonstrated with QR codes in Supplementary Figure 2.

The holographic images of Pin-ball games

We designed another demonstration for programmable metasurface. A video game like Pin-ball game was showed in Supplementary Figure 11. By playing

pre-designed phase profiles sequentially on SLM, 25 frames for a classic pinball game are shown continuously. A ball is moving in a straight line and reflected when it meets a ball-bats. At last, the bat fails to catch the ball and the game is over. This is a simple demonstration to show that our method has great potential in information transportation and in signal modulation. A movie is also shown in the supplementary video for this Pin-ball game.

Figures:

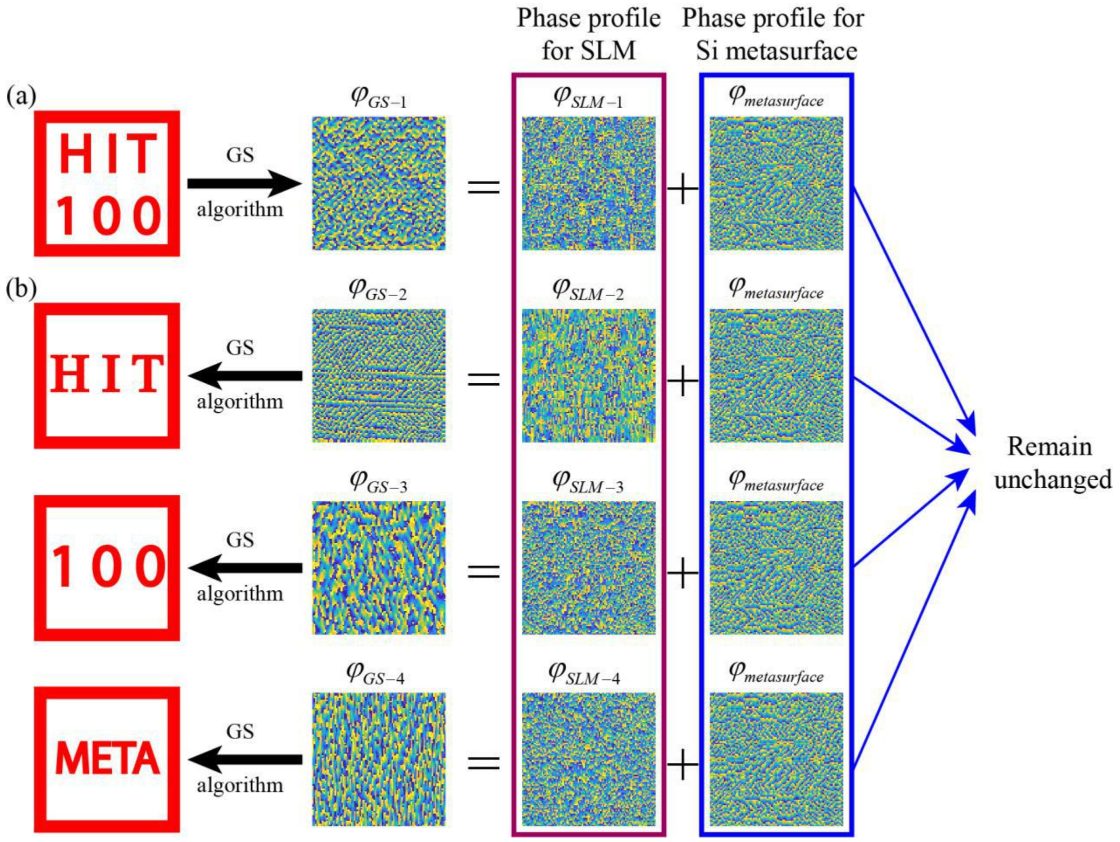

Supplementary Figure 1. **The design process of re-programmable metasurface.** (a) Calculation of phase profile for SLM and metasurface. φ_{GS} is the phase profile calculated from GS algorithm for 'HIT 100' pattern. φ_{SLM} is the separated phase matrix applied onto SLM. $\varphi_{metasurface}$ is the separated phase matrix applied onto metasurface. (b) Through modifying φ_{SLM} and keeping $\varphi_{metasurface}$ unchanged, different image such as 'HIT', '100', 'META' could be constructed.

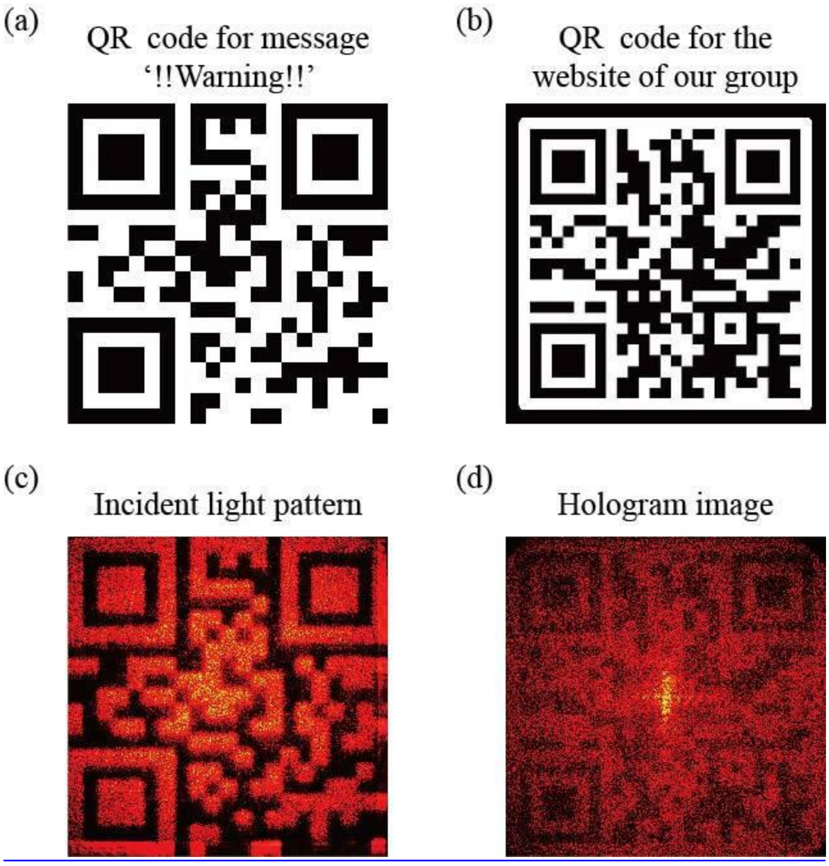

Supplementary Figure 2. The holographic image of QR codes. (a) The designed QR codes for the incident light. (b) The designed output QR codes after passing the metasurface. (c) and (d) are the corresponding experimental results. It is easy to see that the produced QR codes with metasurface are totally different from the incident ones.

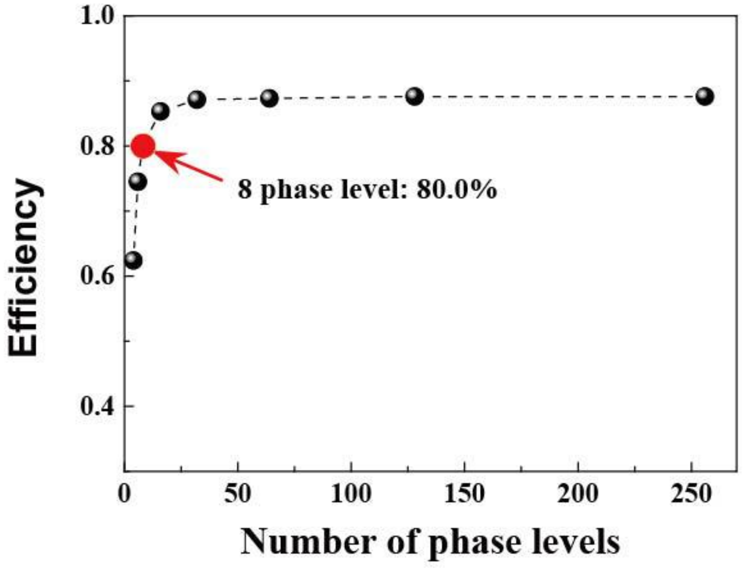

Supplementary Figure 3. The efficiency of meta-hologram as a function of the discrete phase level. When the phase level is 8, the efficiency is above 80%. For the case of > 32, it is the same as the continuous phase. By considering the fabrication difficulty and the efficiency, 8-level phase profile is thus selected.

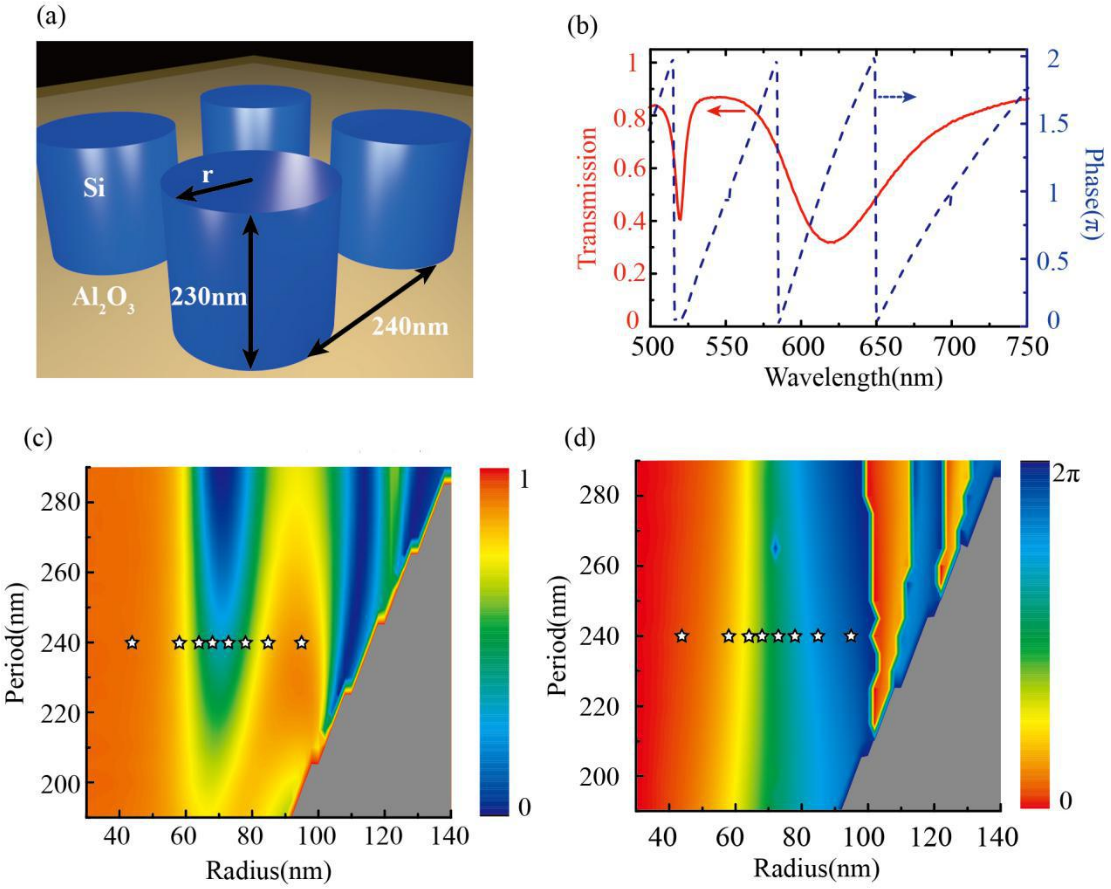

Supplementary Figure 4. Numerical simulation of Si metasurface. (a) Schematic picture of Si nanopillar. (b) Transmission spectrum and phase change of Si metasurface with R = 68nm. (c), (d) are the transmission spectrum and the phase change as a function of period and radius. The right bottom grey area corresponds to the case that the diameter of nanopillar is larger than the lattice size. This is different from the setting in current research and thus is ignored.

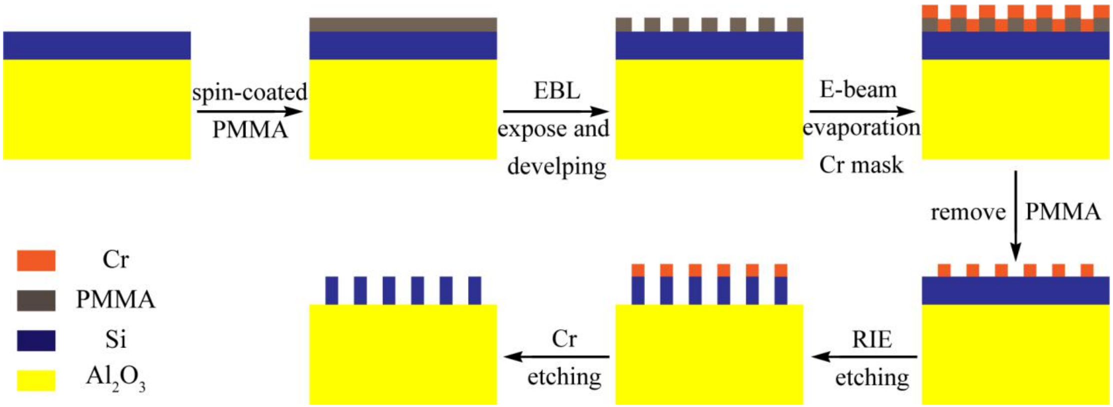

Supplementary Figure 5. The schematic picture of the nano-fabrication process.

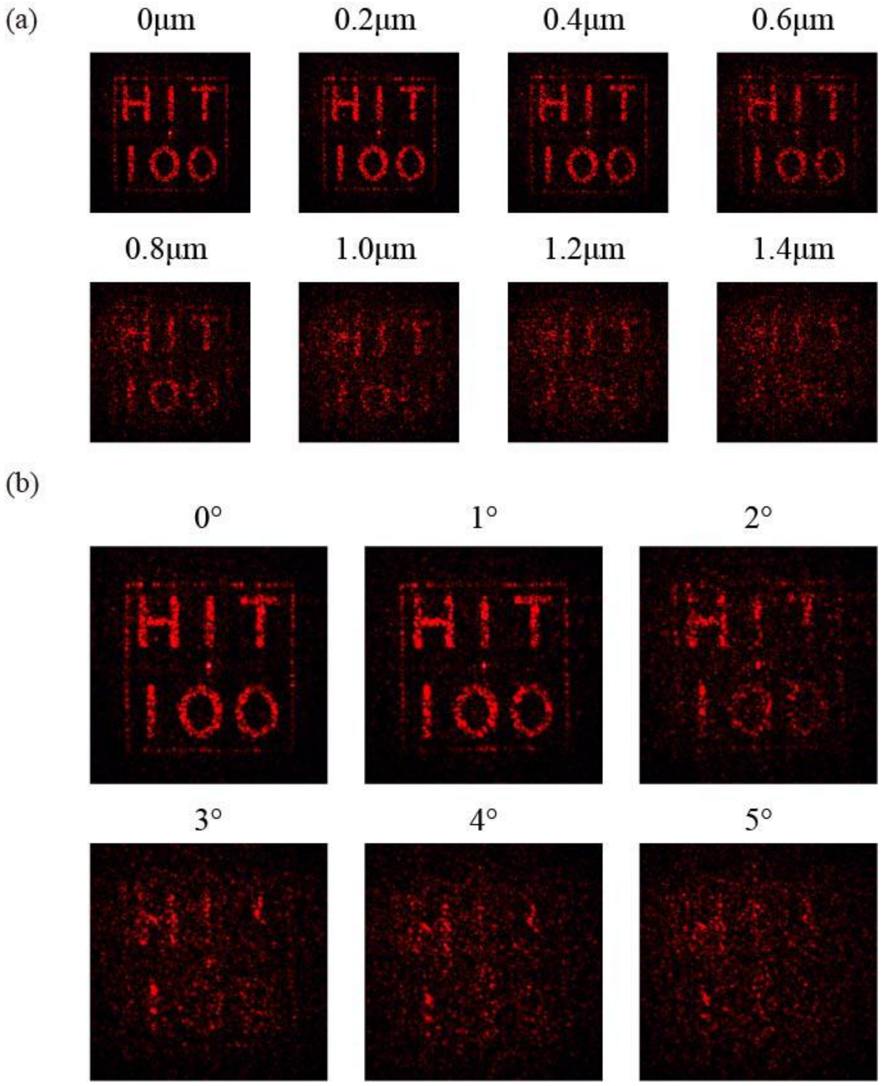

Supplementary Figure 6. Experiment hologram of un-matched initial position. (a) The effect of position deviation to hologram image quality. (b) The effect of angle deviation to hologram image quality.

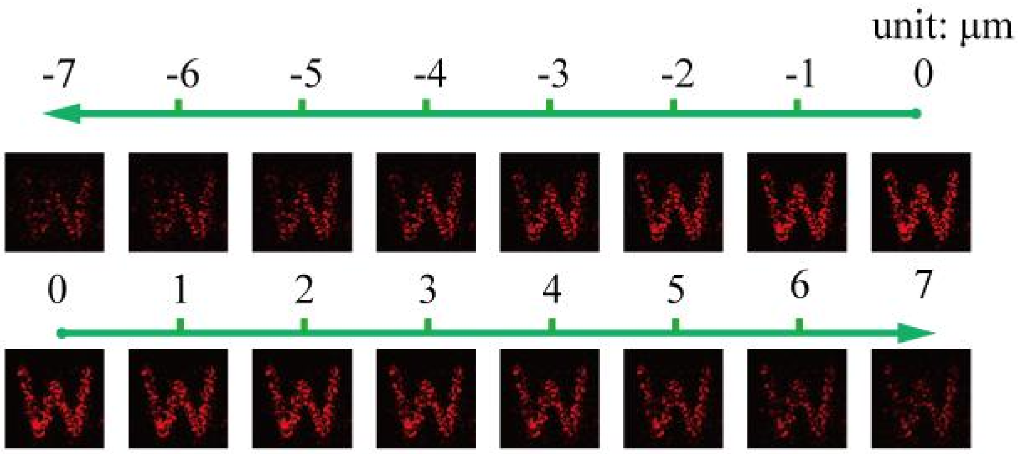

Supplementary Figure 7. Experiment hologram of un-matched longitude position. A ‘W’ shape hologram image under different longitude un-matched position. The number means the distance between sample and perfect focus position. When distance equals to zero, it means that sample was placed in the perfect focus position.

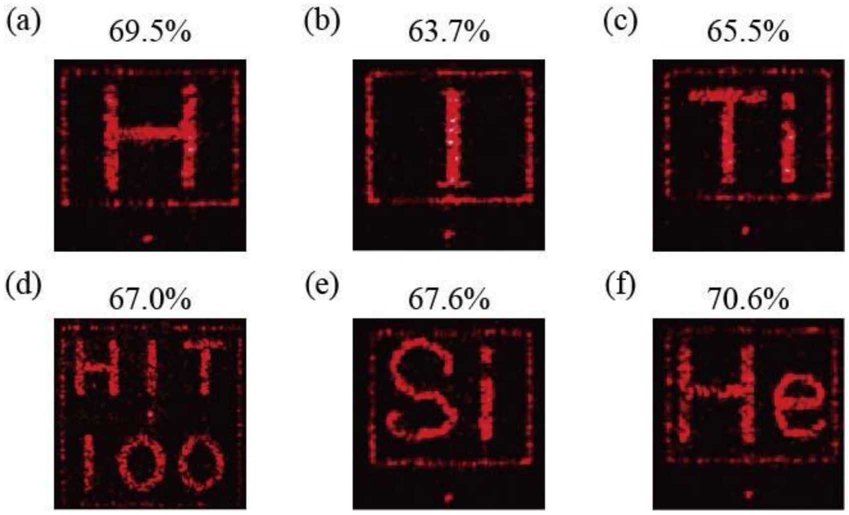

Supplementary Figure 8. Efficiency of several hologram images. Several meta-hologram images and the corresponding efficiency.

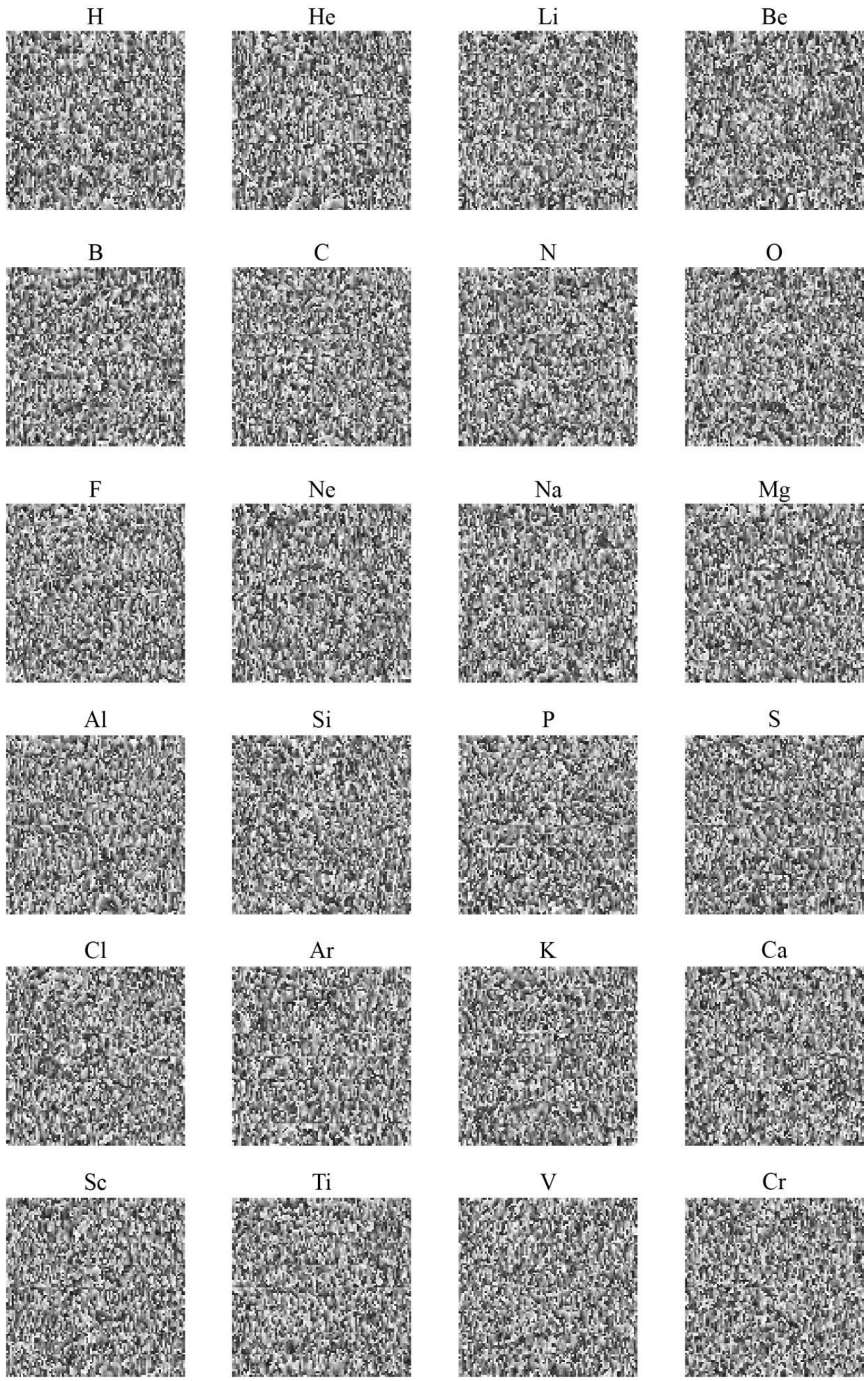
H
He
Li
Be
B
C
N
O
F
Ne
Na
Mg
Al
Si
P
S
Cl
Ar
K
Ca
Sc
Ti
V
Cr

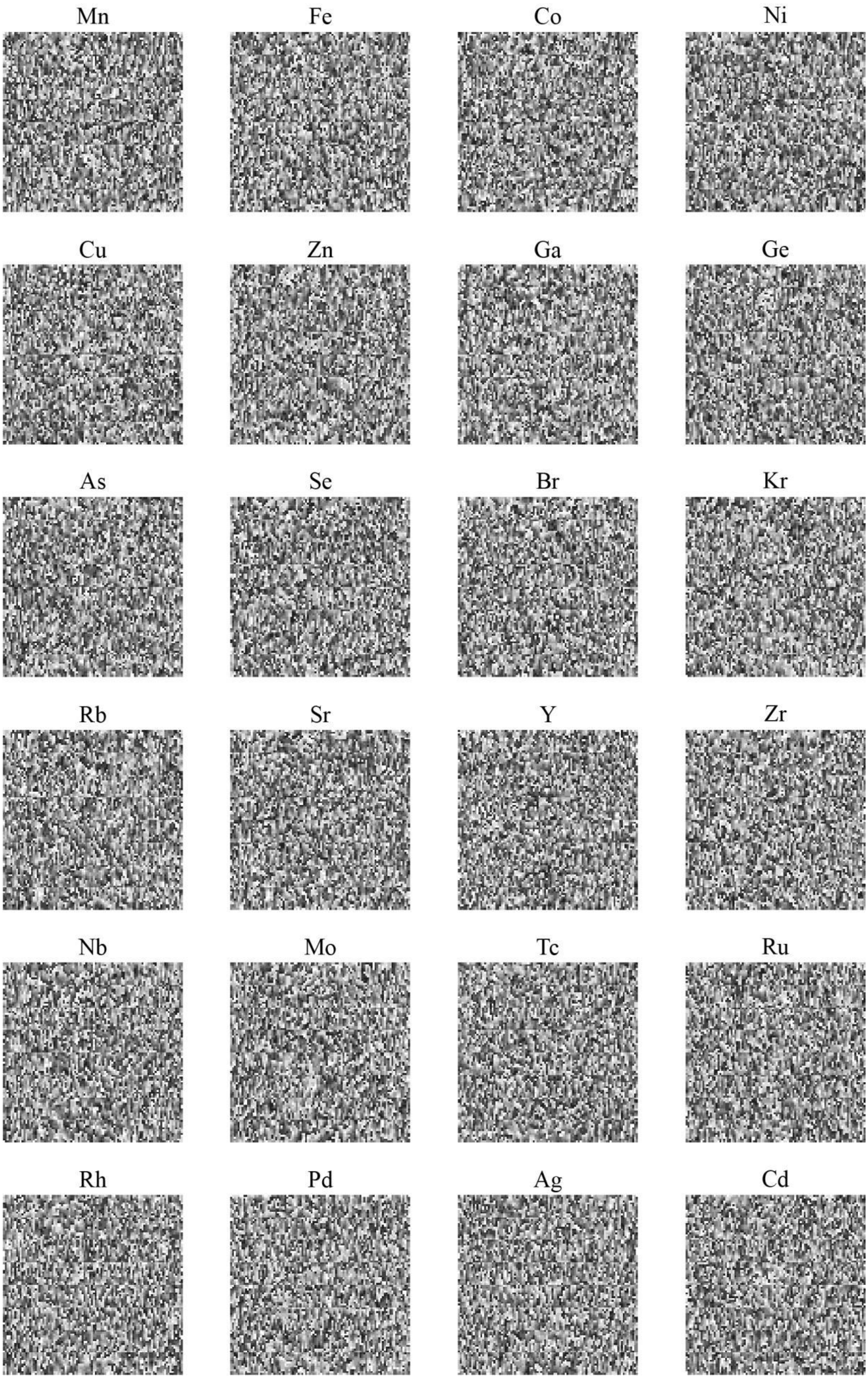
Mn
Fe
Co
Ni
Cu
Zn
Ga
Ge
As
Se
Br
Kr
Rb
Sr
Y
Zr
Nb
Mo
Tc
Ru
Rh
Pd
Ag
Cd

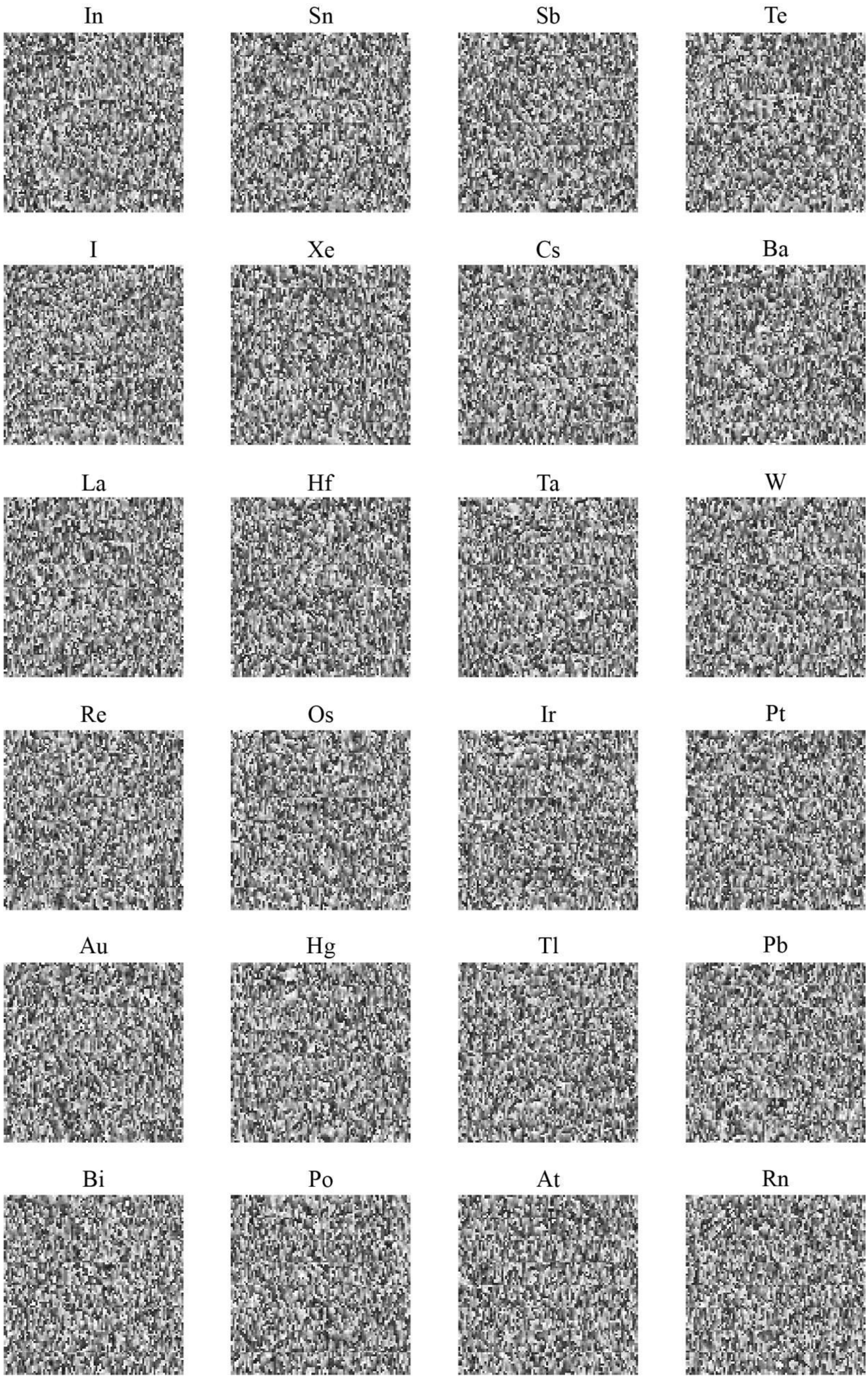
In
Sn
Sb
Te
I
Xe
Cs
Ba
La
Hf
Ta
W
Re
Os
Ir
Pt
Au
Hg
Tl
Pb
Bi
Po
At
Rn

Fr

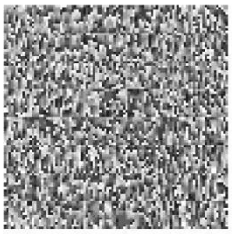

Ra

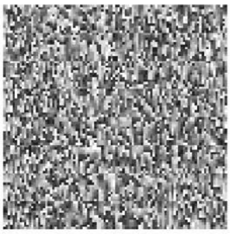

Ac

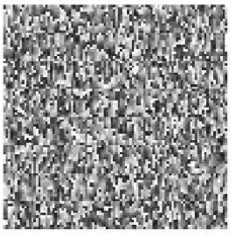

Rf

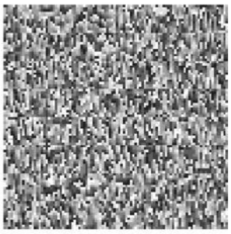

Db

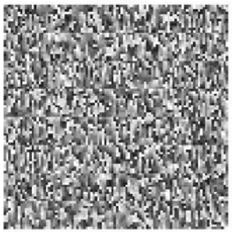

Sg

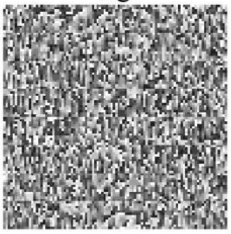

Bh

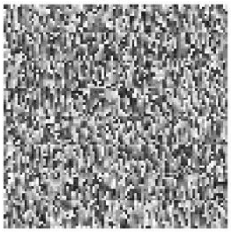

Hs

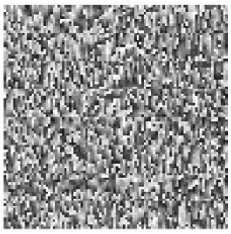

Mt

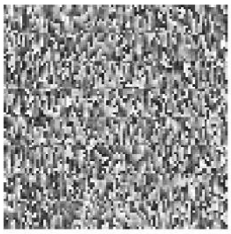

Ds

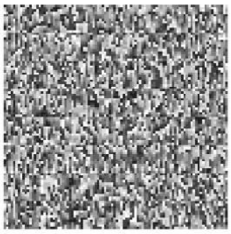

Rg

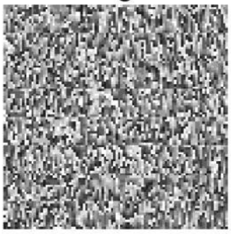

Cn

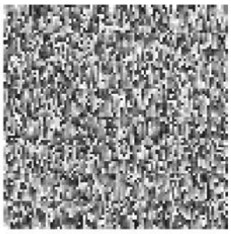

Nh

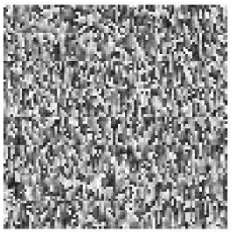

Fl

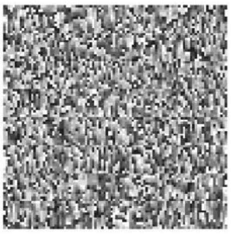

Mc

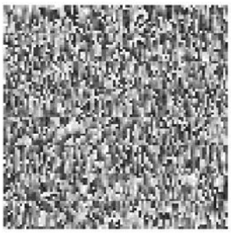

Lv

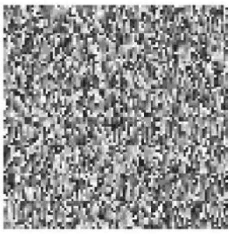

Ts

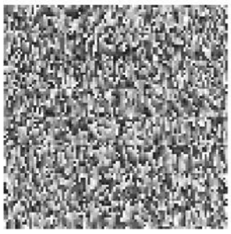

Og

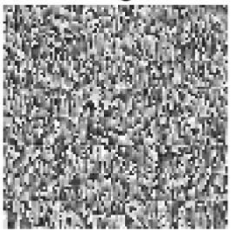

Ce

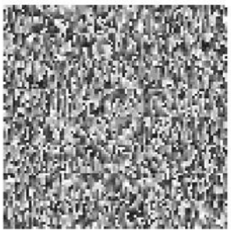

Pr

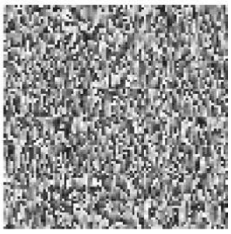

Nd

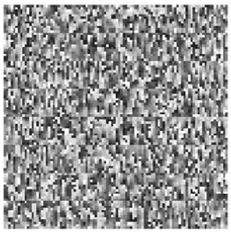

Pm

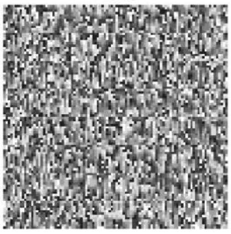

Sm

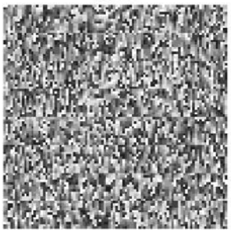

Eu

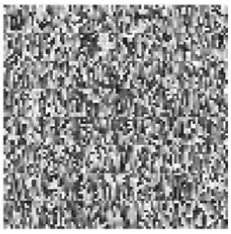

Gd
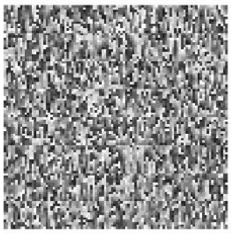
Tb
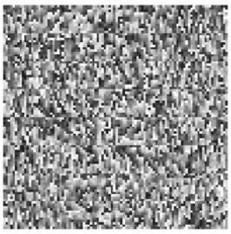
Dy
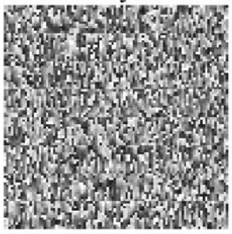
Ho
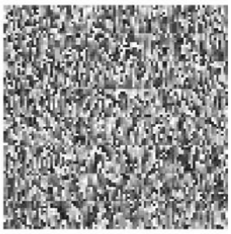
Er
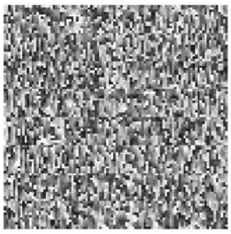
Tm
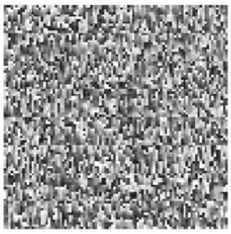
Yb
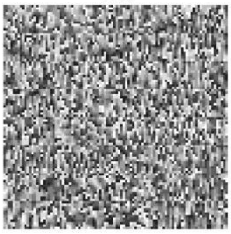
Lu
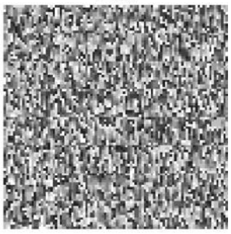
Th
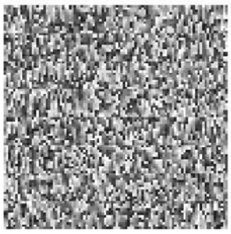
Pa
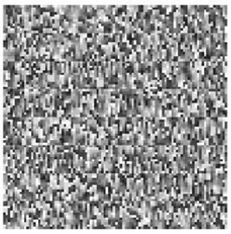
U
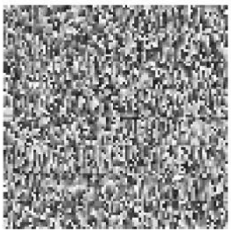
Np
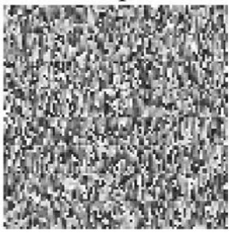
Pu
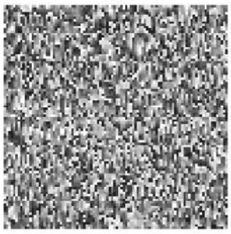
Am
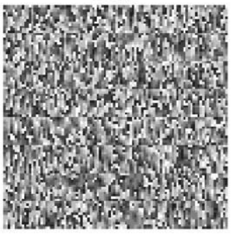
Cm
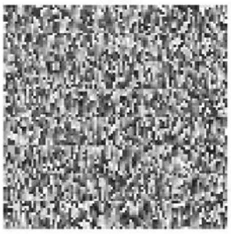
Bk
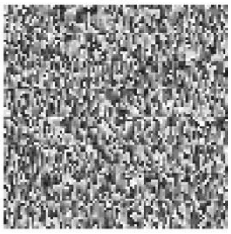
Cf
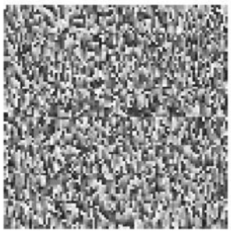
Es
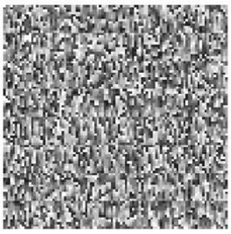
Fm
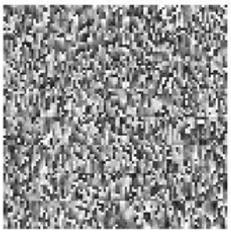
Md
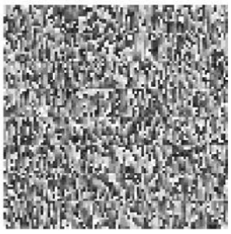
No
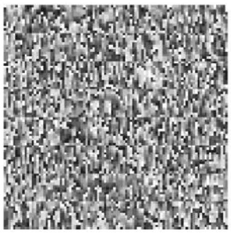
Lr
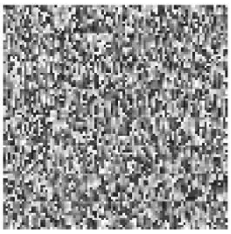

Supplementary Figure 9. The calculated phase profiles of 118 elements. The corresponding hardware Si metasurface is kept as the same one in Fig. 2 of the main text.

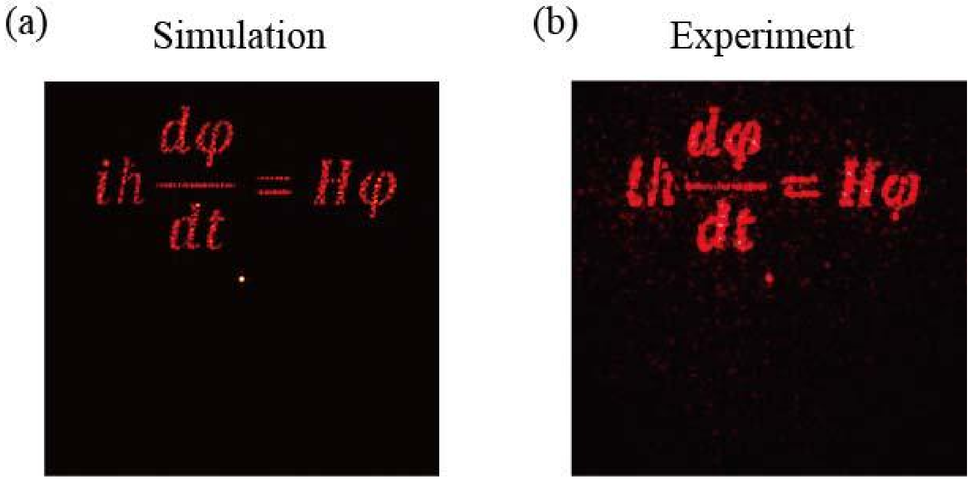

Supplementary Figure 10. The holographic image of the Schrödinger equation. (a) The numerically calculated image. (b) The experimental demonstration. Here the metasurface is the same as the one in the main text and thus the efficiency of the holographic image is also close to the ones in Fig. S8.

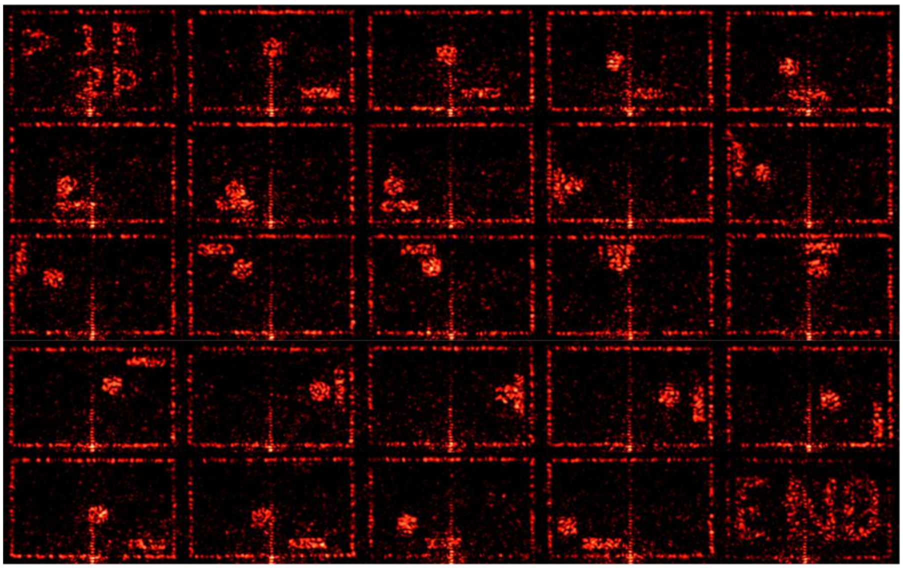

Supplementary Figure 11. The schematic picture of Pin-ball game. Here the metasurface is still the same one as Fig. 2 in the main text.

Reference:

1. Zhou, F., Liu, Y. and Cai, W. Plasmonic holographic imaging with V-shaped nanoantenna array. *Opt. Express* **21**, 4, 4348-4354 (2013).
