# Supplementary material for: Reprogrammable meta-hologram for optical encryption
Source: Nat Commun. 2020 Oct 30;11:5484. doi: 10.1038/s41467-020-19312-9 (PMC7603497; doi:10.1038/s41467-020-19312-9)
Supplement: Supplementary file 2 — Description of Additional Supplementary Files [file 41467_2020_19312_MOESM2_ESM.pdf]

Description of Additional Supplementary Files

File Name: Supplementary Movie 1
Description: The supplementary movie 1 demonstrates a pinball game from start to the end using the metasurface by playing these frames sequentially.
