# Supplementary figures and images for: Reprogrammable meta-hologram for optical encryption
Source: Nat Commun. 2020 Oct 30;11:5484. doi: 10.1038/s41467-020-19312-9 (PMC7603497; doi:10.1038/s41467-020-19312-9)

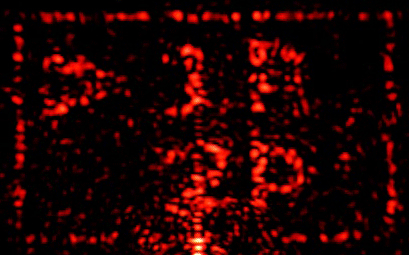

Supplement: Supplementary file 3 — Supplementary Movie 1 [file 41467_2020_19312_MOESM3_ESM.gif]
